# Supplementary material for: Grazing effects on woody and herbaceous plant biodiversity on a limestone mountain in northern Tunisia
Source: PeerJ. 2019 Aug 13;7:e7296. doi: 10.7717/peerj.7296 (PMC6698127; doi:10.7717/peerj.7296)
Supplement: Supplemental Information 1 — Includes Methods not retained in the main text. [file peerj-07-7296-s001.docx]

Supplementary Material

Methods

Identification of plant species

We collected and pressed any plants not identified in the field and compared them with specimens at the herbarium in Tunis or the British Museum herbarium in the United Kingdom. As sampling occurred towards the end of many herbaceous species’ flowering period (Daoud-Bouattour et al. 2007), families like the Liliaceae and Orchidaceae, which die back by early summer, were probably under-recorded. Other species were difficult to identify in this condition, such as *Asphodelus ramosus* subsp. *Ramosus*, whose leaves of resemble those of *Moraea sisyrinchium*, and some other Asphodelaceae. Among other groups, the following species pairs were combined because they could not be distinguished: *Hypochoeris achyrophorus and H. saldensis; Hyoseris radiata* and *H. scabra;* *Hippocrepis minor* and *H. unisiliquosa;* and *Sedum tuberosum* and *S. rubens*.

Since conducting our study, plant nomenclature has changed substantially (Supplementary Tables 1 and 2). We updated all plant names using the Euro-Med Plantbase (PlantBase, 2017) or the Medchecklist (Medchecklist 2012).

Calculation for multiple Olea europea stems:

r_i_ = c_i_/2π

A_i_ = π x r_i_^2^

A = Σ A_i_

r = √ (A/π)

where r_i_ = radius of stem i, c_i_ = circumference of stem i, π = 3.146; A_i_ = surface area of stem i, A = total surface area of all stems and r = equivalent radius.

*Treatment of species data and environmental covariates*

For the environmental covariates we inspected draftsman plots to determine whether variable distributions were approximately linear. We created eight classes of aspect based on compass bearings: (1: 0-45^o^ (NNE); 2: 46-90^o^ (ESE); 3: 91-135^o^ (SSE); 4: 136-180^o^ (SSW); 5: 181-225^o^ (WSW); 6: 226-270^o^ (WNW); 7: 271-315^o^ (NNW); 8: 316-360^o^ (ENE). For the eight aspect classes we grouped variables into an indicator group as this was a binary categorical variable.
