# Supplementary material for: Grazing effects on woody and herbaceous plant biodiversity on a limestone mountain in northern Tunisia
Source: PeerJ. 2019 Aug 13;7:e7296. doi: 10.7717/peerj.7296 (PMC6698127; doi:10.7717/peerj.7296)
Supplement: Supplemental Information 2 — Results included that were not retained in the main text. [file peerj-07-7296-s002.docx]

Results

*Plant species*

We identified 219 of the 348 plant species recorded for Jebel Ichkeul (Fay, 1980). Species richness and diversity were highly variable; high altitude, northerly, ridge crest assemblages were most species-rich, while species-poor sites were prevalent on the heavily grazed and cleared lower southerly slopes, where invading ruderals were abundant. Overall, 21% of sites had more than 50 species, 32% had 40-50 species, 32% had 30-40 species, and the remainder had less than 30 species. The richest site contained 65 species (site 35) and was located near the summit at an altitude of 352 m.a.s.l. Only 16 species were found in the most species-poor quadrat (site 57) which comprised *Ceratonia siliqua* and *Olea europaea* forest. The influence of soil limitations, exposure and grazing in high matorral created clearings rich in herbaceous species, while in deep valleys subject to ephemeral winter flooding closed canopy climax forest had lower species richness.

We found two new species for the National Park reported by (Hollis, 1977), but not by Fay (1980) - *Sideritis romana* L. subsp. *numidica* and *Helminthotheca aculeata*. Species not previously recorded on the Jebel, but present in the plant inventory for the National Park included; *Cichorium intybus,* *Rapistrum rugosum*, *Torilis arvensis*, *Gaudinia fragilis* and *Anogramma leptophylla*. Five new species were found; *Trigonella (arabica*?), *Lathyrus aphaca*, *Hypericum triquetrefolium*, *Stachys* sp. and *Lolium rigidum*.
