# Supplementary material for: Grazing effects on woody and herbaceous plant biodiversity on a limestone mountain in northern Tunisia
Source: PeerJ. 2019 Aug 13;7:e7296. doi: 10.7717/peerj.7296 (PMC6698127; doi:10.7717/peerj.7296)
Supplement: Table S1 — List of woody plant species recorded at Jebel Ichkeul with percentage occurrence at sites. [file peerj-07-7296-s003.docx]

|  |  |  |  |  |  |
| --- | --- | --- | --- | --- | --- |
| Family | Functional group/  life form | Common Name | Scientific Name | % occurrence | Acronym |
| **Woody species** |  |  |  |  |  |
| Fabaceae | Legume | Stinking Bean Trefoil, Mediterranean Stinkbush | *Anagyris foetida* L. | 47.4 | ANAGFOET |
| Fabaceae | Legume |  | *Anagyris* sp. | 2.6 | ANAGSSPS |
| Ericaceae | Other woody | Strawberry Tree | *Arbutus unedo* L. | 9 | ARBUUNED |
| Asteraceae |  | Wormword | *Artemisia arborescens* L. | 2.6 | ARTEARBO |
| Asparagaceae |  | Lesser Asparagus, Wild Asparagus | *Asparagus acutifolius* L. | 21.8 | ASPAACUT |
| Asparagaceae |  | White asparagus | *Asparagus albus* L. | 79.5 | ASPAALBU |
| Fabaceae | Legume | Spiny Broom | *Calicotome villosa* (Poiret) Link | 1.3 | CALIVILL |
| Capparaceae |  | Spineless Caper | *Capparis spinosa* L. | 19.2 | CAPPSPIN |
| Fabaceae | Legume | Carob, St. John's Bread, Locust Bean | *Ceratonia siliqua* L. | 15.4 | CERASILI |
| Cistaceae |  | Pink Rock Rose, Hoary Rock Rose | *Cistus creticus* L. subsp. *eriocephalus* (Viv.) Greuter & Burdet | 3.8 | CISTCRET |
| Cistaceae |  | Montpelier Rock Rose | *Cistus monspeliensis* L. | 9 | CISTMONS |
| Cistaceae |  | Sage-leaved Rockrose | *Cistus salviifolius* L. |  | CISTSALV |
| Ranunculaceae |  | Evergreen Traveller's Joy | *Clematis cirrhosa* L. | 39.7 | CLEMACIRR |
| Ranunculaceae |  | Fragrant clematis | *Clematis flammula* L. |  | CLEMAFLAM |
| Fabaceae | Legume | Mediterranean Crownvetch, Shrubby Crown Vetch | *Coronilla valentina* L. (3 subspecies - not differentiated) | 1.3 | COROVALE |
| Thymelaeaceae |  | Flax-leaved Daphne | *Daphne gnidium* L. | 6.4 | DAPHGNID |
| Ephedraceae |  | Joint-pine | *Ephedra fragilis* Desf.subsp. *fragilis* | 21.8 | EPHEFRAG |
| Ericaceae |  | Tree Heath | *Erica arborea* L. | 5.1 | ERICARBO |
| Ericaceae |  | Mediterranean Heath | *Erica multiflora* L. | 20.5 | ERICMULT |
| Euphorbiaceae |  | Tree Spurge | *Euphorbia dendroides* L. | 14.1 | EUPHDEND |
| Cistaceae |  | Arabian Cistus | *Fumana arabica* L. (Spach) |  | FUMAARAB |
| Cistaceae |  |  | *Fumana laevipes* L. (Spach) | 5.1 | FUMALAEV |
| Plantaginaceae |  | Common or Shrubby Globularia | *Globularia alypum* L. | 6.4 | GLOBALYP |
| Oleaceae |  | Bush Jasmine | *Jasminum fruticans* L. | 71.8 | JASMFRUT |
| Cupressaceae |  | Phoenicean Juniper | *Juniperus phoenicea* L. (2 subspecies - not differentiated) | 5.1 | JUNIPHOE |
| Adoxaceae |  | Evergreen Honeysuckle | *Lonicera implexa* Aiton | 7.7 | LONIIMPL |
| Myrtaceae |  | Common or True Myrtle | *Myrtus communis* L.subsp. *communis* | 5.1 | MYRTCOMM |
| Oleaceae |  | Olive Tree | *Olea europaea* L. subsp. *europaea* var. *europaea* | 94.9 | OLEAEURO |
| Asclepiadaceae |  | African Wolfbane | *Periploca angustifolia* Labill. | 15.4 | PERIANGU |
| Asteraceae |  | Mediterranean Phagnalon | *Phagnalon saxatile* (L.) Cass. |  | PHAGSAXA |
| Oleaceae |  | Mock Privet | *Phillyrea angustifolia* L. | 87.2 | PHILANGU |
| Oleaceae |  | Mock Privet | *Phillyrea latifolia* L. |  | PHILLATI |
| Anacardiaceae |  | Mastic Tree, Lentisk | *Pistacia lentiscus* L. | 93.6 | PISTLENT |
| Anacardiaceae |  | Terebinth, Turpentine Tree | *Pistacia terebinthus* L. | 32.1 | PISTTERE |
| Rhamnaceae |  | Palestine Buckthorn | *Rhamnus lycioides* L. subsp. *oleoides* (L.) Jahandiez & Maire | 23.1 | RHAMLYCI |
| Rubiaceae |  | Wild Madder | *Rubia peregrina* L. | 16.7 | RUBIPERE |
| Asparagaceae |  | Large Butcher's Broom, Spineless Butcher's Broom, Israeli Ruscus | *Ruscus hypophyllus* L. | 5.1 | RUSCHYPO |
| Lamiaceae |  | Pink Micromeria | *Satureja graeca* L. | 10.3 | SATUGRAE |
| Smilacaceae |  | Mediterranean Smilax, Common Smilax, Prickly Ivy | *Smilax aspera* L. | 21.8 | SMILASPE |
| Rhamnaceae |  |  | *Ziziphus lotus* (L.) Lam. subsp. *lotus* | 1.3 | ZIZULOTU |
|  |  |  |  |  |  |
|  |  |  |  |  |  |
