# Supplementary material for: Grazing effects on woody and herbaceous plant biodiversity on a limestone mountain in northern Tunisia
Source: PeerJ. 2019 Aug 13;7:e7296. doi: 10.7717/peerj.7296 (PMC6698127; doi:10.7717/peerj.7296)
Supplement: Table S2 — List of herbaceous plant species recorded at Jebel Ichkeul with percentage occurrence at sites. [file peerj-07-7296-s004.docx]

| Family | Functional group/  growth form | Common Name | Scientific Name | % occurrence | Acronym | |
| --- | --- | --- | --- | --- | --- | --- |
| Poaceae | Graminoid |  | *Agrostis pourretii* Willd. | 5.1 | AGROPOUR | |
| Poaceae | Graminoid |  | *Aira cupaniana* Guss. | 1.3 | AIRACUPA | |
| Amaryllidaceae | Geophyte | Wild Leek | *Allium cf. commutatum* Guss. | 1.3 | ALLICOMM | |
| Amaryllidaceae | Geophyte | Leek | *Allium cf. porrum* L. | 14.1 | ALLIPORR | |
| Amaryllidaceae | Geophyte | Hairy Garlic | *Allium cf. subvillosum* Schultes & Schultes f. | 25.6 | ALLISUBV | |
| Poaceae | Graminoid | Mauritanian Grass | *Ampelodesmos mauritanicus* (Poir.) T.Durand & Schinz | 12.8 | AMPEMAUR | |
| Primulaceae |  | Scarlet Pimpernel | *Anagallis arvensis* L. | 3.8 | ANAGARVE | |
| Fabaceae | Legume | Annual Kidney-vetch | *Anthyllis tetraphylla* L. | 5.1 | ANTHTETR | |
|  |  |  |  |  | (TRIPTETR) | |
| Fabaceae | Legume | Common Kidney Vetch | *Anthyllis vulneraria* L. subsp. *maura* (G. Beck) Maire var. *maura* | 3.8 | ANTHVULN | |
| Araceae | Geophyte | Friar's Cowl | *Arisarum vulgare* Targ. Tozz. | 2.6 | ARISVULG | |
| Xanthorrhoedaceae | Geophyte | Summer Asphodel | *Asphodelus ramosus* L.subsp. *ramosus* | 12.8 | ASPHRAMO | |
| Aspleniaceae |  |  | *Asplenium ceterach* L.subsp. *ceterach* | 10.3 | ASPLCETE | |
| Asteraceae |  | Common Atractylis | *Atractylis cancellata* L.var. *cancellata* | 3.8 | ATRACANC | |
| Poaceae | Graminoid |  | *Avena barbata* Pott ex Link in Schrad., | 26.9 | AVENBARB | |
| Plantaginaceae |  | Mediterranean Bartsia | *Bellardia trixago* (L.) All. | 3.8 | BELLTRIX | |
| Gentianaceae |  | Yellow-wort | *Blackstonia perfoliata* (L.) Huds | 42.3 | BLACPERF | |
| Poaceae | Graminoid |  | Brachypodium distachyum (L.) P.Beauv. | 80.8 | BRACDIST | |
| Poaceae | Graminoid | Large Quaking Grass, Little Quaking Grass | *Briza maxima* L. | 38.5 | BRIZMAXI | |
| Poaceae | Graminoid |  | *Bromus hordeaceus* L. | 3.8 | BROMHORD | |
| Poaceae | Graminoid |  | *Bromus lanceolatus* Roth | 9 | BROMLANC | |
| Campanulaceae |  | Forked Bellflower | *Campanula dichotoma* L. | 20.5 | CAMPDICH | |
| Campanulaceae |  | Small Bellflower | *Campanula erinus* L. | 6.4 | CAMPERIN | |
| Campanulaceae |  | Rampion Bellflower, Rampion | *Campanula rapunculus* L. | 2.6 | CAMPRAPU | |
| Cyperaceae |  |  | *Carex halleriana* Asso subsp. *halleriana* | 9 | CAREHALL | |
| Asteraceae |  | Woolly Carthamus | *Carthamus lanatus* L. | 3.8 | CARTLANA | |
| Poaceae | Graminoid |  | *Catapodium rigidum* (L.) C.E. Hubb. in Dony subsp. *hemipoa* (Delile ex Spreng.) Le Floc’h & Boulos comb. nov. | 65.4 | CATARIGI | |
| Gentianaceae |  |  | *Centaurium candelabrum* H. Lindb. fil. | 6.4 | CENTCAND | |
| Gentianaceae |  | Lesser Centuary | *Centaurium pulchellum* (Swartz) Druce subsp. *pulchellum* | 11.5 | CENTPULC | |
| Arecaceae |  | Dwarf Fan Palm | *Chamaerops humilis* L. | 9 | CHAMHUMI | |
| Pteridaceae |  | Resurrection Fern | *Cheilanthes acrostica* (Balb.) Todaro, | 2.6 | CHEIACRO | |
| Amaranthaceae |  | Nettle-leaved Goosefoot | *Chenopodium murale* L. | 1.3 | CHENMURA | |
| Cuscutaceae |  | Dodder | *Cuscuta epithymum* (L.) L. | 1.3 | CUSCEPIT | |
| Primulaceae | Geophyte | Cyclamen | *Cyclamen africanum* Boiss. Reuter | 5.1 | CYCLAFRI | |
| Boraginaceae |  | Blue Hound's-Tongue | *Cynoglossum creticum* Miller | 3.8 | CYNOCRET | |
| Poaceae | Graminoid | Dogstail grass | *Cynosurus elegans* Desf. subsp *elegans* | 33.3 | CYNOELEG | |
| Poaceae | Graminoid | Orchardgrass, Cocksfoot | *Dactylis glomerata* L. | 14.1 | DACTGLOM | |
| Apiaceae |  |  | *Daucus crinitus* Desf. | 1.3 | DAUCCRIN | |
| Ranunculaceae |  | Short-spurred Larkspur | *Delphinium staphisagria* L. | 2.6 | DELPSTAP | |
| Caryophyllaceae |  | Carnation, Clove Pink | *Dianthus sylvestris* Wulfen subsp. *longicaulis* (Ten.) Greuter & Burdet var. godronianus (Jord.) Kerguélen | 1.3 | DIANSYLV | |
| Dioscoreaceae | Geophyte | Black Bryony | *Dioscorea communis* (L.) Caddick & Wilkin | 1.3 | DIOSCOMM | |
| Asparagaceae | Geophyte | Sea Squill | *Drimia maritima* (L.) Stearn subsp. *maritima* | 3.8 | DRIMMARI | |
| Apiaceae |  | Sea Holly | *Eryngium tricuspidatum* L. | 6.4 | ERYNTRIC | |
| Euphorbiaceae |  | Dwarf Spurge | *Euphorbia exigua* L.subsp. *exigua* var. *exigua* |  | EUPHEXIG | |
| Euphorbiaceae |  | Petty Spurge | *Euphorbia peplus* L. | 16.7 | EUPHPEPL | |
| Caprifoliaceae |  |  | *Fedia caput-bovis* Pomel | 34.6 | FEDICAPU | |
| Apiaceae |  | Giant Fennel | *Ferula communis* L. | 46.2 | FERUCOMM | |
| Poaceae | Graminoid |  | *Festuca coerulescens* Desf. | 5.1 | FESTCOER | |
| Asteraceae |  | Pygmy Cudweed | *Filago pygmaea* L. | 3.8 | FILAPYGM | |
| Asteraceae |  |  | *Filago pyramidata* L. | 2.6 | FILAPYRA | |
| Asteraceae |  | Mediterranean Thistle | *Galactites tomentosa* Moench | 1.3 | GALATOME | |
| Rubiaceae |  |  | *Galium* sp. | 12.8 | GALISPEC | |
| Rubiaceae |  | Southern Cleavers | *Galium verrucosum* Huds.subsp. *verrucosum* | 3.8 | GALIVERR | |
| Poaceae | Graminoid |  | *Gastridium scabrum* C.Presl | 17.9 | GASTSCAB | |
| Geraniaceae |  | Herb Robert | *Geranium robertianum* L. | 25.6 | GERAROBE | |
| Iridaceae | Geophyte | Mediterranean Gladiolus | *Gladiolus communis* L. subsp. *byzantinus* (Mill.) A.P. Hamilton | 1.3 | GLADBYZA | |
| Asteraceae |  | Variable hyoseris | *Hedypnois rhagadioloides* (L.) F.W. Schmidt | 51.3 | HEDYRHAG | |
| Asteraceae |  |  | *Helminthotheca aculeata* (Vahl) Lack | 1.3 | HELMACUL | |
| Fabaceae | Legume |  | *Hippocrepis minor* Munby | 2.6 | HIPPMINO | |
| Brassicaceae |  | Hoary Mustard | *Hirschfeldia incana* (L.) Lagrze-Fossat | 3.8 | HIRSINCA | |
| Poaceae | Graminoid |  | *Hordeum murinum* L. |  | HORDMURI | |
| Asteraceae |  | Perennial Hyoseris | *Hyoseris radiata* L. | 6.4 | HYOSRADI | |
| Hypericaceae |  |  | *Hypericum perfoliatum* L. | 2.6 | HYPEPERF | |
| Asteraceae |  | Mediterranean Catsear | *Hypochaeris achyrophorus* L. | 56.4 | HYPOACHY | |
| Asteraceae |  |  | *Klasea flavescens* (L.) Holub subsp. *mucronata* (Desf.) Cantó & Rivas Mart. | 3.8 | KLASFLAV | |
| Apiaceae |  | Kundmannia | *Kundmannia sicula* (L.) D.C. (?) | 2.6 | KUNDSICU | |
| Poaceae | Graminoid | Hare's Tail Grass | *Lagurus ovatus* L. subsp. *ovatus* | 12.8 | LAGUOVAT | |
| Poaceae | Graminoid |  | *Lamarckia aurea* (L.) Moench | 1.3 | LAMAAURE | |
| Fabaceae | Legume | Yellow Vetchling | *Lathyrus aphaca* L. | 1.3 | LATHAPHA | |
| Fabaceae | Legume | Crimson Pea | *Lathyrus clymenum* L.subsp. *articulatus* (L.) Ball | 7.7 | LATHCLYM | |
| Linaceae |  | Upright Flax | *Linum strictum* L. subsp. *spicatum* (Pers.) Nyman | 10.3 | LINUSTRI | |
| Linaceae |  |  | *Linum cf. usitatissimum* L. | 11.5 | LINUUSIT | |
| Asteraceae |  |  | *Lonas annua* (L.) Vines & Druce | 61.5 | LONAANNU | |
| Fabaceae | Legume | Creta Birdsfoot Trefoil | *Lotus creticus* L. | 17.9 | LOTUCRET | |
| Fabaceae | Legume | Edible Birdsfoot Trefoil | *Lotus edulis* L. | 1.3 | LOTUEDUL | |
| Apiaceae |  |  | *Magydaris pastinacea* (Lam.) Paol | 2.6 | MAGYPAST | |
| Malvaceae |  | Southern Mallow | *Malva nicaeensis* All. | 1.3 | MALVNICA |  |
| Fabaceae | Legume | Prickly Medick | *Medicago intertexta* subsp. *ciliaris* (L.) Ponert | 1.3 | MEDIINTE |  |
| Fabaceae | Legume | Round-fruited Medick | *Medicago orbicularis* (L.) Bartal. | 3.8 | MEDIORBI |  |
| Fabaceae | Legume |  | *Medicago secundiflora* Durieu | 1.3 | MEDISECU |  |
| Fabaceae | Legume | Medick sp. | *Medicago* sp. | 1.3 | MEDISPEC |  |
| Poaceae | Graminoid |  | *Melica cupanii* Guss. | 3.8 | MELICUPA |  |
| Caryophyllaceae |  |  | *Minuartia hybrida* (Vill.) Schischk. | 9 | MINUHYBR |  |
| Plantaginaceae |  | Lesser Snapdragon | *Misopates orontium* (L.) Raf | 1.3 | MISOORON |  |
| Ranunculaceae |  | Love-in-a-mist | *Nigella damascena* L. | 47.4 | NIGEDAMA |  |
| Fabaceae | Legume |  | *Ononis alba* Poiret | 33.3 | ONONALBA |  |
| Fabaceae | Legume | Bird Restharrow | *Ononis ornithopodioides* L. | 1.3 | ONONORTH |  |
| Orchidaceae | Geophyte |  | *Ophrys scolopax* Cav. | 2.6 | OPHYSCOL |  |
| Cactaceae |  |  | *Opuntia ficus-indica* (L.) Mill. | 1.3 | OPUNFICU |  |
| Asparagaceae | Geophyte | Lesser Star of Bethlehem | *Ornithogalum umbellatum* L. | 2.6 | ORNIUMBE |  |
| Orobanchaceae |  | Branched Broomrape | *Orobanche ramosa* L. | 9 | OROBRAMO |  |
| Poaceae | Graminoid | Smilo Grass | *Oryzopsis (Piptatherum) miliacea* (L.) Benth. & Hook. f. ex Asch. & Schweinf | 2.6 | ORYZMILI |  |
| Fabaceae | Legume | Garden Pea | *Pisum sativum* subsp. *elatius* (MB.) Ascherson & Graebner | 2.6 | PISUSATI |  |
| Plantaginaceae |  | Toothed Plantain | *Plantago serraria* L. | 3.8 | PLANSERR |  |
| Polypodiaceae | Geophyte | Southern Polypody | *Polypodium cambricum* L. subsp.*cambricum* | 2.6 | POLYCAMB |  |
| Lamiaeceae |  | White Hedge-nettle | *Prasium majus* L. | 21.8 | PRASMAJU |  |
| Asteraceae |  | Mediterranean Fleabane | *Pulicaria odora* (L.) Rchb. | 3.8 | PULIODOR |  |
| Ranunculaceae | Geophyte | Autumn Buttercup | *Ranunculus bullatus* L. subsp. *bullatus* | 1.3 | RANUBULL |  |
| Ranunculaceae | Geophyte | Hairy Buttercup | *Ranunculus sardous* Crantz subsp. *xatardii* (Lapeyr.) Rouy & Fouc. in Rouy | 2.6 | RANUSARD |  |
| Ranunculaceae | Geophyte |  | *Ranunculus spicatus* Desf. subsp. *maroccanus* (Coss.) Greuter & Burdet | 9 | RANUSPIC |  |
| Brassicaceae |  | Annual Bastard Cabbage | *Rapistrum rugosum* (L.) All. | 1.3 | RAPIRUGO |  |
| Resedaceae |  | White Mignonette | *Reseda alba* L. subsp. *alba* | 1.3 | RESEALBA |  |
| Poaceae | Graminoid |  | *Rostraria cristata* (L.) Tzvelev | 21.8 | ROSTCRIS |  |
| Polygonaceae |  |  | *Rumex thyrsoides* Desf. | 1.3 | RUMETHYR |  |
| Saxifragaceae |  | Rue-leaved Saxifrage | *Saxifraga tridactylites* L. | 2.6 | SAXATRID |  |
| Asteraceae |  | Common Golden Thistle | *Scolymus hispanicus* L. | 5.1 | SCOLHISP |  |
| Fabaceae | Legume | Many-flowered Scorpiurus | *Scorpiurus muricatus* L. | 10.3 | SCORMURI |  |
| Asteraceae |  |  | *Scorzonera undulata* Vahl. | 2.6 | SCORUNDU |  |
| Scrophulariaceae |  | Nettle-leaved Figwort | *Scrophularia peregrina* L. | 12.8 | SCROPERE |  |
| Crassulaceae |  | Azure Stonecrop | *Sedum caeruleum* L. | 11.5 | SEDUCAER |  |
| Crassulaceae |  |  | *Sedum pubescens* Vahl | 9 | SEDUPUBE |  |
| Crassulaceae |  |  | *Sedum tuberosum* Coss. & Letourn. | 3.8 | SEDUTUBE |  |
| Selaginellaceae |  | Tooth-leaved Clubmoss | *Selaginella denticulata* (L.) Spring | 21.8 | SELADENT |  |
| Rubiaceae |  | Field Madder | *Sherardia arvensis* L. | 19.2 | SHERARVE |  |
| Lamiaceae |  | Common Sideritis | *Sideritis romana* L. subsp. *numidica* Batt. | 15.4 | SIDEROMA |  |
| Caryophyllaceae |  | Dense-flowered Catchfly | *Silene bellidifolia* Juss. ex Jacq. | 2.6 | SILEBELL |  |
| Caryophyllaceae |  | Pink Pirouette, Dwarf Pink Star | *Silene colorata* Poir. | 3.8 | SILECOLO |  |
| Asteraceae |  | Mediterranean Sow Thistle | *Sonchus tenerrimus* L. | 11.5 | SONCTENE |  |
| Lamiaceae |  |  | *Stachys ocymastrum* L. (Briq) | 3.8 | STACSPEC |  |
| Caryophyllaceae |  | Common Chickweed | *Stellaria neglecta* Weihe | 9 | STELNEGL |  |
| Poaceae | Graminoid |  | *Stipa capensis* Thunb. | 1.3 | STIPTORT |  |
| Apiaceae |  | Spreading Hedge Parsley | *Torilis arvensis* (Huds.) Link | 11.5 | TORIARVE |  |
| Apiaceae |  | Knotted Hedge Parsley | *Torilis nodosa* (L.) Gaertn. | 17.9 | TORINODO |  |
| Fabaceae | Legume | Narrow-leaved Clover | *Trifolium angustifolium* L.subsp. *angustifolium* | 7.7 | TRIFANGU |  |
| Fabaceae | Legume |  | *Trifolium arvense* L. | 24.4 | TRIFARVE |  |
| Fabaceae | Legume |  | *Trifolium campestre* Schreb | 50 | TRIFCAMP |  |
| Fabaceae | Legume | Rough Clover | *Trifolium scabrum* L. | 25.6 | TRIFSCAB |  |
| Fabaceae | Legume | Starry Clover | *Trifolium stellatum* L. | 11.5 | TRIFSTEL |  |
| Crassulaceae | Geophyte | Wall navelwort | *Umbilicus rupestris* (Salisb.) Dandy | 5.1 | UMBIRUPI |  |
| Urticaceae |  | Roman Nettle | *Urtica pilulifera* L. | 1.3 | URTIPILU |  |
| Rubiaceae |  | Hairy Valantia | *Valantia hispida* L. | 1.3 | VALAHISP |  |
| Rubiaceae |  | Wall Valantia | *Valantia muralis* L. | 2.6 | VALAMURA |  |
| Valerianaceae |  | Crowned Corn Salad | Valerianella discodia (L.) Loisel. | 1.3 | VALECORO |  |
| Valerianaceae |  | Hairy-fruited or Italian Corn Salad | *Valerianella eriocarpa* Desv. subsp. *eriocarpa* | 6.4 | VALEERIO |  |
| Plantaginaceae |  | Grey Field-speedwell | *Veronica polita* Fries | 11.5 | VEROPOLL |  |
| Fabaceae | Legume | Common Vetch | *Vicia sativa* L. | 3.8 | VICISATI |  |
| Fabaceae | Legume | Smooth Tare | *Vicia tetrasperma* (L.) Schreber | 1.3 | VICITETR |  |
| Poaceae | Graminoid |  | *Vulpia ligustica* (All.) Link | 47.4 | VULPLIGU |  |
| Poaceae | Graminoid | Brome Fescue | *Vulpia* sp. | 3.8 | VULPSPEC |  |
