# Supplementary figures and images for: Grazing effects on woody and herbaceous plant biodiversity on a limestone mountain in northern Tunisia
Source: PeerJ. 2019 Aug 13;7:e7296. doi: 10.7717/peerj.7296 (PMC6698127; doi:10.7717/peerj.7296)

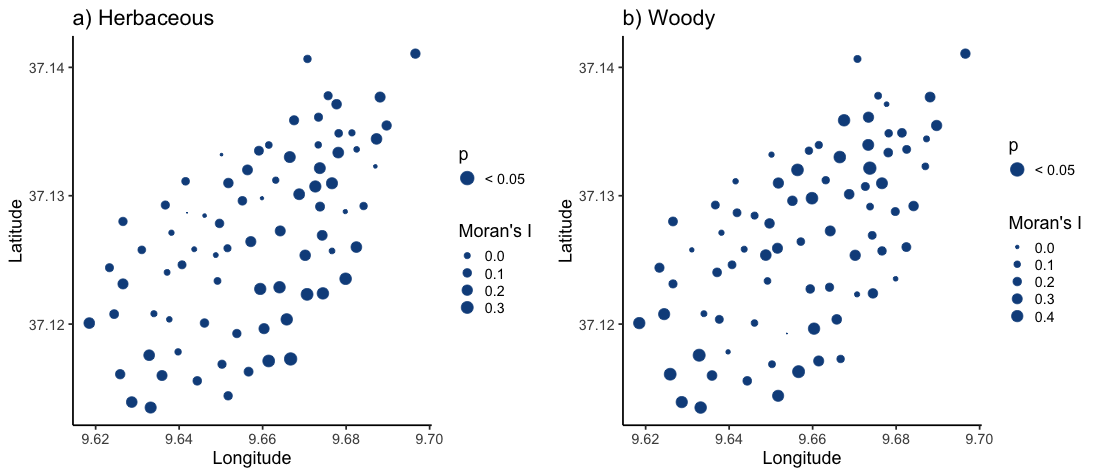

Supplement: Figure S1 — Point size is scaled according to Moran’s I values. Blue points are statistically significant (p < 0.05), indicating positive spatial autocorrelation between sites (note: all points were statistically significant). [file peerj-07-7296-s005.png]

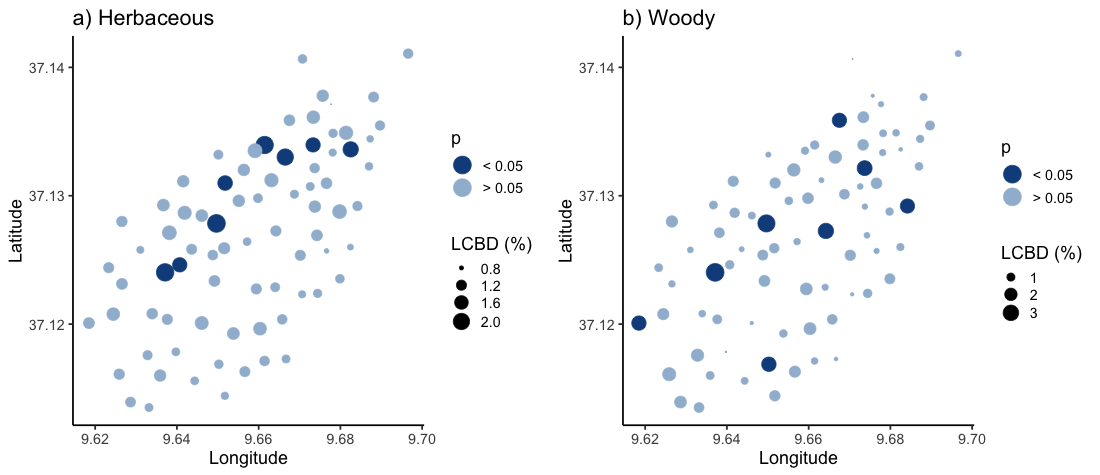

Supplement: Figure S2 — Point size is scaled according to LCBD values (%). Point colour reflects statistical significance, where dark blue indicates statistical significance (p < 0.05), while light blue indicates non-significance (p > 0.05). [file peerj-07-7296-s006.png]
