# Supplementary material for: Grazing effects on woody and herbaceous plant biodiversity on a limestone mountain in northern Tunisia
Source: PeerJ. 2019 Aug 13;7:e7296. doi: 10.7717/peerj.7296 (PMC6698127; doi:10.7717/peerj.7296)
Supplement: French translation of abstract [file peerj-07-7296-s007.docx]

Effets de pâturage sur la biodiversité des boisés et des herbacés sur une montagne calcaire au nord de la Tunisie.

L’Abstrait:

Le pâturage est une problématique de gestion et de conservation de la forte diversité des maquis méditerranéens. Nous avons échantillonné les plantes ligneuses et herbacées séparément sur une montagne calcaire avec de forts gradients mésico-xériques dans le Parc national de l’Ichkeul, en Tunisie. Nous avons testé l’effet de la pression de pâturage, évaluée sur une échelle de 1 à 3, sur l'abondance et la composition des plantes avant et après avoir contrôlé l’effet de variables environnementales. Nous avons trouvé que les sites au bord du lac sur le visage mésique de la montagne ont une composition d’espèces plus unique comparée aux autres sites, et que les phorbes ont contribué le plus fortement à la diversité bêta herbacée sur la montagne. Nous avions ensuite séparé les effets combinés et individuels de l’environnement abiotique, pâturage, l’activité humaine, et la configuration spatiale sur la diversité bêta des plantes herbacées et ligneuses, par le biais de partitionnement de variance. Cependant, l'effet individuel du pâturage sur la composition des communautés ne peut être séparée de l’effet de la configuration spatiale des sites, en raison de la distribution spatiale de régime de pâturage sur la montagne.

Fait important, nous avons constaté que les communautés herbacées et ligneuses réagissaient différemment aux différentes intensités de pâturage: la diversité bêta des herbacées était plus élevée entre les sites sans pression de pâturage, tandis que la diversité bêta ligneuse a atteint son maximum lors d’un pâturage léger. La composition de la communauté herbacée était sensible à toute intensité de pression de pâturage, et une pression de pâturage modérée à élevée a entrainée l'homogénéisation biotique entre communautés. D’autre part, la composition de la communauté ligneuse est restée semblable entre les sites sous aucune ou sous une légère pression de pâturage, mais différait sous un pâturage modéré à intense. Une analyse de la variance par permutation a démontré que le pâturage avait un effet significatif sur la composition des communautés, après avoir contrôlé l’effet des variables abiotiques et spatiales. Nos résultats offrent un aperçu de l’effet du pâturage sur la végétation maquis de Jebel Ichkeul, agissant comme un microcosme de problématiques en conservation et en gestion de la biodiversité ailleurs en Méditerranée. Nous suggérons qu'une combinaison de surveillance et de pâturage contrôlée pourrait maintenir et promouvoir la diversité des plantes afin de préserver la biodiversité de la végétation maquis de la région, dans le but de potentiellement maintenir un refuge climatique vital pour les espèces endémiques vulnérables. Fait important, notre étude fournit une base de référence utile sur la communauté végétale de Jebel Ichkeul avec laquelle comparer les changements de végétation futurs.
